# Comparison of Hybrid Enthalpy–Porosity Models in the Analysis of Solute Macro-Segregation in Binary Alloy Centrifugal Casting

**DOI:** 10.3390/ma18245632

**Published:** 2025-12-15

**Authors:** Mirosław Seredyński, Jerzy Banaszek

**Affiliations:** Institute of Heat Engineering, Faculty of Power and Aeronautical Engineering, Warsaw University of Technology, Nowowiejska Str. 21/25, 00-665 Warsaw, Poland; miroslaw.seredynski@pw.edu.pl

**Keywords:** centrifugal alloy casting, chemical inhomogeneity, thermo-solutal convection, multi-scale numerical simulation, identification of different grain structure zones, front tracking method

## Abstract

This paper presents the detailed comparisons of solute macro-segregation pictures predicted by different meso-macroscopic simulations, based on the single-domain enthalpy–porosity approach coupled with distinct models of flow resistance in the two-phase zone. In the first, the whole zone is treated as a Darcy’s porous medium (EP model); in the other two, the columnar and equiaxed grain structures are distinguished using either the coherency point (EP-CP model) approach or by tracking a virtual surface of columnar dendrite tips (EP-FT model). The simplified 2D model of a solidifying cast in a centrifuge is proposed, and calculations are performed for the Pb-48wt. % Sn cast at various hypergravity levels and rotation angles. It is shown, in the example of Sn-10wt. % Pb alloy, that the predicted macro-segregation strongly depends on the mesoscopic model used, and the EP-FT simulation (validated with the AFRODITE benchmark) provides the most realistic solute inhomogeneity pictures. The EP-FT model is further used to investigate the impact of the hyper-gravity level and the cooling direction on the compositional nonuniformity developing in centrifuge casting. The hyper-gravity level visibly impacts the macro-segregation extent. The region of almost uniform solute distribution in the slurry zone rises with the increased effective gravity, though the solute channeling is more severe for higher gravity and rotation angles. A-channeling and V-channeling were observed for angles between the gravity vector and cooling direction lower than 120° and higher than 120°, respectively.

## 1. Introduction

One of the major flaws of the alloy cast solidifying under terrestrial gravity is its chemical inhomogeneity, known as solute macro-segregation. This phenomenon is attributed to the solute segregation at the individual grains’ scale due to different solute solubility in the liquid and solid phases and then to convective transport of the excess solute, the solidification shrinkage, and the relative motion of the liquid and solid phases at the whole cast’s scale. Thus, the evolution of a non-uniform distribution of the solute concentration and highly solute-reach long and narrow channels, which post-solidification thermal manufacturing processes can hardly remove, considerably deteriorates the quality of alloy casting products.

In this context, manufacturing of metal alloy casts in the centrifugal field has attracted foundry engineers and researchers, as the high-speed rotation enhances melt flow and significantly alters the processes of heat and solute transfer at both the grain’s and the whole cast’s scales, thus changing the developing dendrite structures, solute distributions, and the macro-segregation pictures. Therefore, for the last decades, vast experimental research has been conducted all over the world to comprehend better the role of strength and directions of centrifugal forces in complex transport processes occurring in metal alloy solidification and to search for appropriate conducting of the centrifugal casting process to obtain the product of satisfactory grain structure and mechanical properties.

From the research papers [1,2,3,4,5,6,7,8] and several others available in the worldwide literature involving in situ and real-time observations of various metal alloy samplings solidifying in the hyper-gravity field, it comes out that final grain structures and thermo-mechanical properties of the cast are subject to many mold and processing factors, such as cast dimensions, direction and speed of rotation and temperature gradient, the solidification rate, the composition of the molten alloy and others. So, for a high-quality product of hyper-gravity casting, it is essential to tailor this specialized manufacturing method by continuously monitoring and optimizing its process parameters.

For this purpose, a complete understanding and detailed knowledge of multi-scale heat and mass transfer processes are needed, including the role of external forces in developing chemical inhomogeneity in the final product of centrifugal casting. However, such a complete study is not possible only through cumbersome, expensive, and time-consuming experiments. Several advanced models of computer simulation of heat and mass transfer processes occurring at the scale of individual grains (the mesoscopic scale) and the whole casting product (the macro-scale) have been developed in the last two decades to assist this cognitive endeavor significantly.

Computer simulations at the mesoscopic scale of individual grains, based on the phase-field method (e.g., [9,10]), Cellular Automaton and the Lattice Boltzmann methods (e.g., [11]), show that the melt flow modulated under the hyper-gravity conditions significantly affects the grain nucleation, interface instability, constitutional undercooling, the primary dendrite arms spacing (PDAS) and growth of columnar and equiaxed crystals, their migration and the phenomenon of Columnar–to-Equiaxed Transition (CET), and thus gives the potential for grain refinement.

But the chemical heterogeneity at the scale of individual grains, inherent to the difference in solubility at the liquid–solid interface and non-equilibrium solidification, while associated with the relative motion of the liquid and solid phases, (caused by thermosolutal convection, solidification shrinkage, grains sedimentation) leads to the solute macro-segregation developing in a whole cast domain containing an enormous number of grains. Here, computer simulation models used at the grain’s scale would require a tremendous and unavailable burden of computer resources nowadays. Therefore, meso-macroscopic modeling has been developed, where key calculations are performed on the macroscopic scale, and information about the developing grains’ structures is transferred to the macroscopic model through the effective properties of the two-phase region, the so-called mushy zone. Several such multi-scale computer simulation models have been proposed in the last three decades to predict compositional macro-segregation of chemical species in terrestrial gravity conditions. Macroscopic conservation equations of mass, momentum, energy, and species concentration are based on the mixture theory (e.g., Bennon and Incropera [12], Ni and Incropera [13]) or volume averaging (e.g., Beckermann and Viskanta [14]).

The mushy zone is formed in non-isothermal alloy solidification, where the competing columnar and equiaxed/globular grain structures develop. Motionless columnar dendrites are immersed in a molten alloy; in the other part of the mushy zone, equiaxed/ globular grains are suspended or/and transported in the alloy melt, and the CET can develop in the proceeding solidification. Various models of the mushy zone’s permeability have been proposed and supplemented to the macroscopic calculations of the transport equations. Some researchers treat the whole two-phase region as Darcy’s porous medium using the Kozeny–Carman model of permeability (e.g., Založnik and Combeau [15], Kumar et al. [16], Cao et al. [17]). Li et al. [18] simulated macro-segregation during directional solidification of TiAl ingots in the external magnetic field. They applied the enthalpy–porosity model, based on the two-phase mixture approach, and calculated it on a 2D finite element mesh. They treated the entire mushy zone as a columnar porous medium, where equiaxed crystals were absent. Xu et al. [19] employed a volume-averaged mixed columnar–equiaxed enthalpy–porosity model for the solidification of a titanium-based alloy in a strong electromagnetic field. They have shown that although the melt flow induced by thermo-solute natural convection is visibly weaker than that resulting from electromagnetic forces, buoyancy convection remains crucial for the developing spatial pattern of solute macro-segregation. However, they assumed that the mushy zone, with a solid volume fraction range of 0.01–0.9, consists of only columnar dendrite grains and the liquid melt.

The others distinguish zones of columnar and equiaxed grains, where different models of the resistance to melt flow are applied. Recently, Xu et al. [20] used the Eulerian meso-macroscopic model where the liquid, columnar dendrite, and equiaxed grain phases were treated separately to analyze the macro-segregation pictures in an Al-4.34 wt.% Cu billet. They applied two different criteria for separating the columnar and equiaxed regions in the mushy zone. The equiaxed grains were trapped by the columnar ones when the sum of solid fractions of both these dendritic structures exceeded the value of 0.637 or when the volume fraction of columnar phase rose over 0.2.

In many research papers, these two different grain structures are identified by the inspection of the solid fraction value in the mushy zone (e.g., Ilegbusi and Mat [21], Vreeman and Incropera [22], Krane [23], Zhang et al. [24]). When it exceeds the dendrite coherency point (DCP) value, Darcy’s flow model with the permeability concept is used; otherwise, the pseudo-viscosity [17] or semi-solid medium [21] models are applied. Alternatively, Banaszek and Seredyński [25] have proposed the replacement of the DCP model used in the identification of different dendritic structures by the direct tracking of a virtual surface, being a locus of the columnar dendrite tips and moving according to the local law of crystal growth on a fixed non-structural control-volume mesh. They have compared both models in the prediction of the solute macro-segregation pictures of Pb-Sn alloys solidifying in the rectangular cavities under the terrestrial gravity and concluded that the predicted channel segregates are prone to the used control-volume mesh, the applied permeability formula, and the method of separating different dendritic structures within the mushy zone (Seredynski and Banaszek [26]).

However, the research reported so far on computer simulations of transport processes accompanying the centrifugal casting is less extensive than that concerning the process in terrestrial gravity conditions. It is mainly concerned with the pouring process; only a few research papers have addressed the issue of the effect of the strength and direction of hyper-gravity on the solute inhomogeneity developing throughout the whole cast at different stages of the centrifugal casting.

Daloz et al. [27] have developed a 2D axisymmetric multi-scale computer simulation model for the centrifugally cast Al-Ti cylindrical sample. The proposed simulation is based on the volume-averaged Euler–Euler two-phase approach, where macroscopic thermal and solute convection calculations are coupled with microscopic nucleation and crystal growth models. Based on the analysis of the strength of terrestrial gravity, Coriolis, and centrifugal forces, only the latter has been taken into account in the calculations. The coherency point approach has been used to distinguish the zones of stationary columnar dendrites and moving equiaxed grains. Battaglioli et al. [28] have developed a 2D computer simulation for predicting the influence of gravity strength on the evolution of fluid flow, temperature, and grain structures during the directional solidification of Ti-Al alloy on a centrifuge. The model is based on a single set of transport equations in the solid–liquid mixture, discretized on a structural control-volume mesh in the plane perpendicular to the rotation axis. The front tracking procedure is applied to trace the developing columnar dendrites zone, where the porous medium model is exploited. This research study omitted consideration of solute convection, and macro-segregation was not analyzed. Pan et al. [29] have developed a 3D macroscopic model for the centrifugal investment casting of large-sized titanium alloy components. The proposed computer simulation is based on discretizing macroscopic mass, momentum, and energy transport equations on a non-conformal finite difference mesh. The 3D numerical simulation of the directional columnar solidification of Ti-Al alloys (consistent with the configuration of ESA GRADECET experiments) is presented by Fernández et al. in [30,31]. The applied volume-averaging model accounts for thermo-solutal convection, centrifugal, and Coriolis accelerations when the temperature gradient along the cylindrical sample is antiparallel to the total apparent gravity (sum of centrifugal and terrestrial gravity). The Kozeny–Carman hydrodynamic permeability of the columnar mushy zone and Boussinesq’s model for thermo-solutal buoyancy forces have been adopted. The research is, however, restricted only to the development of the columnar dendrites during directional solidification. Lv et al. [6] have presented the numerical analysis of the mold-filling process and solidification of a wedge-shaped Al-Cu cast under horizontal centrifugation.

Their micro-macroscopic model adopted the cellular automaton method to calculate the grain nucleation, which was coupled with the finite element calculations of macroscopic transport processes. The presented parametric analysis concentrated only on the impact of centrifugal forces on the cast’s microstructure changes and confirms that the increase in rotation speed and the higher cooling rate cause the reduction in grain average sizes and the secondary dendrite arm spacing.

The combined experimental research and computer simulations have led to significant progress in understanding the role of enhanced melt flow in a centrifuge. Despite these two-decade efforts, more still has to be performed to achieve a better quantitative understanding of complex multi-scale processes deciding about grains’ micro and macro structures of hyper-gravity casting products. Among still open scientific questions, there is the influence of the interaction between the enhanced flow of melt and the Coriolis acceleration on convection and the growth of solidification microstructures [9,10,32], the mechanisms behind columnar crystals progress [4], and the macro-segregation developing at different hyper-gravity casting stages [5,30,32]. Also, since numerical modeling is a primary potential source of valuable information on enhancing the reliability of the whole casting process, improving the accuracy and trustworthiness of multi-scale computer simulations of solute segregation under the centrifugal and Coriolis forces remains a challenge. The enhanced mesoscopic simulations [9,10,11] give a deep insight and much better understanding of the impact of the strength and orientation of hyper-gravity and the augmented melt flow on the PDAS, the growth of single and multiple columnar and equiaxed dendritic crystals, and the other micro and mesoscopic characteristics of the mushy zone. On the other hand, the above-discussed filling process and mechanical defects (e.g., [6,27,29]), and the directional solidification with the imposed thermal gradient direction (e.g., [29]), do not take into account the terrestrial gravity (e.g., [27]), Coriolis acceleration (e.g., [26,27]), the thermo-solutal buoyancy (e.g., [7,27,28,29]) and the different dendritic structures and the CET developing in the mushy zone (e.g., [30,31]).

So efforts should be continued to develop further advanced computer simulations involving more accurate predictions of the solute macro-segregation and channeling phenomena under hyper-gravity, where the melt flow process is more complex and changeable than in gravity casting, and thus to frame reliable correlations between hyper-gravity forces and the resulting grain structures and chemical and mechanical properties of the centrifugal casting product.

The presented paper is the authors’ contribution to this current research area, in which the issue of the role of the assumed model of mushy zone in predicting reliable pictures of solute macro-segregation in hyper-gravity is discussed. For this purpose, three different meso-macroscopic simulations, based on the single-domain enthalpy–porosity approach, are compared, each coupled with distinct models of flow resistance in the mushy zone. In the first, the whole two-phase zone is treated as a Darcy’s porous medium (EP model); in the other two, the columnar and equiaxed grain structures are distinguished using either the coherency point (EP-CP model) approach or by tracking a virtual surface of columnar dendrite tips (EP-FT model). The last one, being an extension of the EP-FT model proposed in [25], is used for the first time in a detailed study of solute inhomogeneity under hyper-gravity conditions. For these comparisons, a simplified 2D model of the representative rotating plane is developed based on a thorough analysis of all forces acting on a cast in the centrifuge and their projections onto the plane. Having shown that the EP-FT model is the most reasonable choice for a reliable simulation of complex macro-segregation pictures in centrifugal alloy casting, it has been further used to address the interesting issue of the impact of the hyper-gravity level and the cooling direction on the development of the macro-segregation pictures, and the tendency to form freckles in solidifying Pb-Sn alloy casting.

The paper is organized as follows. In Section 2, the mathematical and computational model in the form of transport equations, boundary and initial conditions, closure relations, geometry, and material properties, is detailed. The emphasis is placed on the description of buoyancy volumetric forces inherent in centrifugal casting, as well as specific transport models in the slurry and porous zones, and the front tracking procedure. In Section 3, the credibility analysis is carried out. It consists of model validation with the AFRODITE experiment, and simulations are executed for the Sn-10wt% Pb alloy solidification at purely natural convection conditions and terrestrial gravity. In the second part of Section 3, the mesh sensitivity analysis is performed for the solidification of Pb-48wt. % Sn alloy, at an enhanced gravity level (5 g), and in the rotated frame. The parametric analysis (Section 4) consists of several steps that highlight the various aspects of the model. In Section 4.1, the models (EP, EP-FT, and EP-CP) are examined, and the impact of gravity level, cooling orientation, and coherency point value (EP-CP) on the development of macro-segregation and the formation of segregation channels is considered. In Section 4.2, the EP-FT model is investigated. The role of orientation and gravity level in the macro-segregation picture is analyzed. Final conclusions are presented in Section 5.

## 2. Mathematical and Computational Model

Macroscopic calculations of binary alloy centrifugal solidification are often built on the single continuum enthalpy–porosity model utilizing the classical two-phase mixture theory (e.g., [12,13]). During the cooling and solidification of an alloy’s cast, the two-phase zone (mushy zone) evolves, usually containing two distinguished regions of columnar and equiaxed grains. The former, having a strong directional nature, appears as a dense crystalline-like matrix filled with inter-dendritic liquid. In the equiaxed zone, grains freely grow, mutually influence, and move in the melt, and the CET phenomenon might occur. To account for different resistances to the melt flow occurring within these two diverse dendritic structures, Darcy’s porous medium model is commonly used in the columnar dendrite zone, whereas the slurry medium model is applied to the equiaxed grain zone. Therefore, it is necessary to distinguish regions of different grain structures in the computational model before applying distinct descriptions of the flow conditions. Two methods have been used to identify grain regions of varying flow conditions; they are coupled with the enthalpy–porosity model. The first one is the commonly used coherency point approach (e.g., [22,23,24]), and the computational model is further referred to as the EP-CP (Enthalpy Porosity–Coherency Point). The second one, further called the EP-FT (Enthalpy Porosity–Front Tracking), involves the front tracking procedure to trace the envelope of columnar dendrite tips on finite volume meshes (e.g., [25,26,29,33,34,35]). The macro-segregation and channeling pictures developing in the centrifugal investment casting predicted by the EP-CP and EP-FT models are compared in this research.

### 2.1. Macroscopic Conservation Equations

A single set of the macroscopic conservation equations of mass, momentum, energy, and the solute transport in the liquid–solid mixture appears as

(1) $$\nabla \cdot \left(\rho V\right)=0,$$

(2) $$\begin{array}{ll} \frac{\partial \left(\rho V\right)}{\partial t} & +\nabla \cdot \left(\rho VV\right)=\nabla \cdot \left(\mu_{L}\nabla V\right)-\nabla \cdot \left(\mu_{L}f_{S}\nabla V_{S}\right)\\ & +\nabla \cdot \left(\mu_{S}f_{S}\nabla V_{S}\right)-\nabla \cdot \left[\left(\frac{\rho f_{S}}{f_{L}}\right)\left(V-V_{S}\right)\left(V-V_{S}\right)\right]+F_{v}\;\\ & -\nabla p+\left(f_{S}\nabla p\right)Ṽ-\mu_{L}f_{L}K^{-1}VṼ \end{array},$$

(3) $$\frac{\partial \left(\rho cT\right)}{\partial t}+\nabla \cdot \left(\rho cVT\right)=\nabla \cdot \left(k\nabla T\right)+\frac{\partial \left(f_{S}\rho L\right)}{\partial t}-\nabla \cdot \left(f_{L}\rho LV_{L}\right),$$

(4) $$\begin{array}{ll} \frac{\partial }{\partial t}\left(\rho C\right) & +\nabla \cdot \left(\rho VC\right)=\nabla \cdot \left(\rho D\nabla C\right)+\nabla \cdot \left(\rho f_{L}D_{L}\nabla \left(C_{L}-C\right)\right)\\ & +\nabla \cdot \left(\rho f_{S}D_{S}\nabla \left(C_{S}-C\right)\right)-\nabla \cdot \left[\rho \left(V-V_{S}\right)\left(C_{L}-C\right)\right] \end{array},$$

**V**, T, and C are the velocity vector, temperature, and solute concentration, respectively. Thermo-physical properties: ρ, k, c, μ, L, and D denote density, thermal conductivity, specific heat, dynamic viscosity, latent heat of fusion, and solute mass diffusivity, respectively. Indices S and L refer to the solid and liquid phases. g and f are phase volumetric and mass fractions, respectively, and stand for the switching function, which equals 1 in the columnar dendrites zone and 0 elsewhere.

According to the mixture theory, the velocity, thermal conductivity, overall solute concentration, and solute mass diffusivity of the liquid–solid mixture are given by the following:

(5) $$V=f_{S}V_{S}+f_{L}V_{L},$$

(6) $$k=g_{S}k_{S}+g_{L}k_{L},$$

(7) $$C=f_{S}C_{S}+f_{L}C_{L},$$

(8) $$D=f_{S}D_{S}+f_{L}D_{L},$$

where C_L_ and C_S_ are the average solute concentrations, and D_L_ and D_S_ are the solute diffusivities in the liquid and solid phases, respectively.

The volumetric sources, **F**_V_, in Equation (2) are given by

(9) $$\begin{array}{ll} F_{V} & =-g_{L}\rho_{L,ref}[\beta_{T,L}(T-T_{ref})+\beta_{C,L}(C_{L}-C_{L,ref})]\\ & \;\;\;\;\;\;\;\cdot \left\{g-\omega \times \left(\omega \times R\right)-2\left(\omega \times V\right)\right\} \end{array},$$

The first and second terms in the square brackets refer to thermal and solute buoyancy forces described by the commonly used Boussinesq’s natural convection model. In a stationary coordinate system, they are multiplied by the terrestrial gravity acceleration vector, **g**, and constitute the gravity force, **F**_g_. The second and third terms in the curly brackets express the centrifugal, **F**_cf_, and Coriolis, **F**_C_, forces.

### 2.2. Simplified 2D Geometrical Model of the Volumetric Forces

The volumetric forces acting on a liquid volume in the rotating frame are three-dimensional; the terrestrial gravity works in the vertical direction, centrifugal acceleration acts radially concerning the centrifuge axis, and the Coriolis force operates in the horizontal planes.

**Figure 1:**
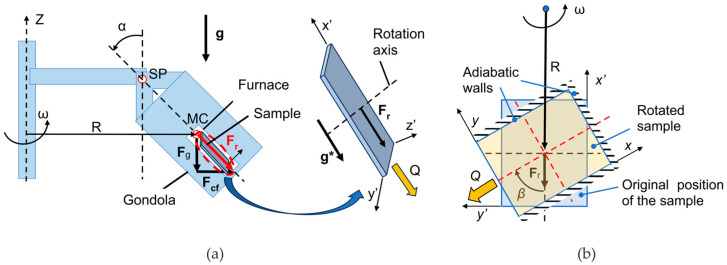
The schematic of the centrifuge arrangement: (**a**) the rotating gondola with the furnace and sample, and the magnified view of the sample and the direction of heat dissipation at the reference sample position (right side, indicated by the arrow); (**b**) the sample orientation in the gondola after rotation in the x’-y’ plane.

The gondola with the furnace and the sample, mounted at the end of the rotating arm (Figure 1), rotates with the angular velocity, ω, around the axis Z. The rotation angle of the gondola, α, depends on the ratio of the centrifugal force and the gravity force and can be derived with the formula

(10) $$\alpha =\arctan\left(\frac{\left|F_{cf}\right|}{\left|F_{g}\right|}\right)=\arctan\left(\frac{\omega^{2}R}{g}\right),$$

the resulting (acceleration) force, **F**_r_, is the sum of the centrifugal, **F**_cf_, and gravity forces, **F**_g_, and is applied to the mass center of the gondola.

The rotation rate, ω, and the distance from the axis, R, are adjusted to ensure the required gravity acceleration level. Three values of gravity acceleration are considered: 1 g, 5 g, and 15 g. For the first one, treated as the reference case, the rotation speed is obviously equal to 0.

In the presented study, centrifugal casting in a simplified layout is considered. The sample is positioned in the center of the gondola. The cuboid shape of the mold is assumed. The basic orientation of the sample is presented in Figure 1, according to the Cartesian coordinate system x’, y’, z’ (Figure 1a), fixed with the gondola, rotating around axis Z. The length of the edge parallel to the z’ axis is much lower than other dimensions of the cavity, so the 2D geometry of the computational domain is assumed. Additionally, the interactions of the molten alloy with walls parallel to the x’-y’ plane are omitted, and the fluid velocity components in the z’ directions are set to zero.

In this paper, the relation between the direction of cooling and the resultant acceleration force is investigated, so various orientations of the sample are considered. To accomplish various heat dissipation directions, given with Q, the sample is rotated around the axis perpendicular to the x’-y’ plane and crossing the central point of the sample, marked as the rotation axis in Figure 1b. For each case the rotation angle, β, is measured between the modified direction of the dissipated heat transfer, Q, and the resulting force, **F**_r_, as presented in Figure 1b and it is considered as a multiple of a 30-degree angle. The limiting values of angles 0° and 180° correspond to the cooling from the bottom and top, respectively. The size of the considered domain is 0.1 m in the x-axis direction and 0.06 m in the y-axis direction (Figure 1b. The new coordinate system, namely x, y, z, describes the orientation of the system relative to the acceleration force.

The first and second terms in the curly brackets in Equation (9) refer to the gravitational acceleration and the centrifugal forces, respectively, and their influence can be described with the resulting (acceleration) force, **F**_r_.

The Coriolis force (the last term in Equation (9)) may act outside of the plane x-y, so, to account for the impact of that force in the considered 2D geometry, it is necessary to take the components of the Coriolis force cast in the x-y plane. However, for the highest enhanced gravity acceleration, 15 g, and the assumed process parameters, ω = 30 rad/s, R = 2.42 m, and assumed maximum molten alloy velocity Vmax = 0.1 m/s, the Coriolis force magnitude is comparable to the gravity force, and 15 times lower than the resulting force. So, in the simplified analysis, neglecting the Coriolis force is justified.

### 2.3. Identification of Zones of Different Grain Structures

Two different approaches are used to identify zones of prevailing distinct grain structures within the mushy zone. The first one is the dendrite coherency point model (DCP), which is based on continuously examining a solid fraction value in the mushy zone during developing solidification. Darcy’s flow model with the permeability concept is used when the solid fraction exceeds a critical value, generally accepted as equal to the solid fraction at the temperature at which the growing dendrites begin to impinge upon each other and form the stationary coherent dendritic network. Otherwise, in the zone where small equiaxed dendrites float in the alloy melt, the slurry model is adopted. This approach has been commonly used in meso-macroscopic computer simulations of metal alloy solidification under terrestrial gravity (e.g., [21,22,23,24,25,26]) and by Daloz et al. [27] in the numerical analysis of solute segregation in the centrifugally cast Al-Ti cylindrical sample. According to this methodology, the porous and slurry regions are identified with the switching function, Ṽ_coh_, which depends on the solid fraction according to the following formula (e.g., Ilegbusi and Mat [21]):

(11) $${Ṽ}_{coh}=0.5+\frac{1}{\pi }\tan^{-1}[100(g_{S}-g_{S,coh})],$$

where g_s,coh_ is the volumetric fraction of the solid phase when the grains start to impinge.

The main uncertainty arising when using the DCP model concerns the reliable estimation of the packing solid fraction value at the dendrite coherency point. The literature review shows that because of the lack of experimental data for various metal alloys, some authors misuse the DCP value determined experimentally by Arnberg et al. [36] for only aluminum-based alloys, while others set its value arbitrarily. This parameter is also known as the coherency solid fraction (e.g., Ilegbusi and Mat [21]), and is referred to as g_s,coh_ in this paper. The DCP solid fraction, g_s,coh_, strongly depends on the alloy composition (e.g., [36,37]), and its assumed value has a significant impact on numerically predicted solute concentration fields and macro-segregation pictures (e.g., [23,24]). Moreover, Banaszek and Seredyński [25] have shown that the use of the isoline of constant solid packing fraction for distinguishing different grain structures is inaccurate, particularly at early stages of solidification when the formation of channel segregates develops much earlier than the CET appears, i.e., for smaller values of the solid fraction.

An alternative method for identifying zones of different dendritic structures is based on direct tracking of a virtual surface, being a locus of the columnar dendrite tips and moving on a fixed control-volume mesh during proceeding solidification according to the assumed crystal growth kinetics. This approach, originated by Browne and Hunt [33] for the case of purely diffusive alloy solidification, has been further extended to modeling columnar and equiaxed binary alloy solidification driven by natural thermal convection [34] and thermo-solutal convection [35] on 2D structural control-volume meshes. Recently, Seredyński and Banaszek [26] have generalized the front tracking procedure to 2D unstructured control-volume meshes and used it to analyze the role of different permeability laws and various microstructure characteristic lengths on the numerically predicted solute macro-segregation developing during solidification of Pb-Sn alloys in the terrestrial gravity conditions. Battaglioli et al. [28] have applied the front tracking method in their 2D computer simulation of centrifugal directional solidification of Ti-Al alloy to study the role of hyper-gravity on the evolution of fluid flow, temperature, and grain structures. Their model is, however, restricted to a structural control-volume mesh and does not involve the solute transport, so the solute macro-segregation is not addressed.

In the presented study, for the first time to the authors’ best knowledge, the front tracking approach, coupled with the macroscopic description of heat, momentum, and solute mass transport phenomena driving the binary alloy solidification, is used to address the issue of the role of hyper-gravity acceleration in the development of chemical inhomogeneity in a solidifying cast.

**Figure 2:**
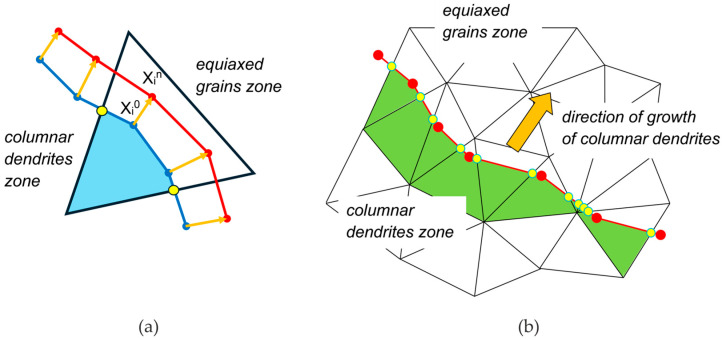
The schematic of (**a**) the front tracking procedure on a fixed unstructured control-volume mesh; (**b**) determination of the switching function. The blue and red dots relate to the positions of markers at the previous and next time steps, respectively. The yellow dots present the intersections of the linear segments with the faces of the control volume mesh and are necessary to determine the volume of the cell behind the front.

The main novelty of the EP-FT simulation model lies in tracking the envelope of columnar dendrite tips on a fixed control-volume unstructured mesh at the end of each subsequent calculation cycle of the transport processes conducted using the enthalpy–porosity (EP) model. This hypothetical interface of columnar dendrite tips is represented by a series of linear segments connected with massless markers, as shown in Figure 2a. An i-th marker’s initial location, $X_{i}^{0}$, is known from the previous time step; the marker moves in the normal direction to the front towards the bulk liquid, according to the determined local undercooling, ΔT, and the assumed columnar dendrite growth law. The marker’s new position is determined as $\;X_{i}^{n}=X_{i}^{0}+\;n\cdot V_{tip}\cdot \Delta t$ and a new shape of the front is obtained by the interpolation between new locations of the markers (red line segments in Figure 2a). In the presented calculations, the columnar dendrite tip velocity, V_tip_, is assumed to be independent of the acceleration level and is determined using the Kurz–Giovanola–Trivedi law [38] and the least-square approximation to obtain $V_{tip}=3.964\cdot 10^{-7}\Delta T^{2.568}$ for the further analyzed Pb-48wt.% Sn alloy.

The front-position-based switching function, Ṽ_ft_, is related to the position of the columnar front and is defined at a consecutive time step as the ratio of the part of the control volume marked with green color to the whole corresponding control volume (Figure 2b). Control volumes crossed by the front have properties of both the slurry and porous media. The source terms in the momentum balance equation, Equation (2), are activated in this region with the appropriate weights depending on the Ṽ_ft_ function. Based on the actual position of the columnar front, the Ṽ_ft_ is equal to zero in the bulk liquid and the slurry zone and to one in the fully porous medium. It smoothly changes between these limit values along the control volume crossed by the columnar front. The function Ṽ_ft_ is also used to calculate actual values of the phase velocities as follows:

(12) $$V_{L}=\frac{V}{f_{L}+\left(1-Ṽ\right)\cdot \left(1-f_{L}\right)}\;;\;\;\;\;\;\;\;\;\;V_{S}=\left(1-Ṽ\right)V_{L},$$

where for parameter Ṽ, the coherency switching function, Ṽ_coh_, or the front-position-based switching function, Ṽ_ft_, can be substituted.

More detailed information on procedures related to tracking of the front and its relation with emerging structures can be found in our previous papers, e.g., Seredynski and Banaszek [26].

The initial position of the front is coincident with the cooled walls. In the case presented in Figure 1, only one wall is cooled, located at x = 0, where x and y coordinates are related to the system rotated by angle β. The beginning of the front is positioned at the point (x, y) = {0.0, 0.06} and the end at the point (x, y) = {0.0, 0.0}.

### 2.4. Transport Properties and Closure Relationships

The system of macroscopic transport equations (Equations (1)–(4)) is based on the mixture theory approach, where a two-phase medium is treated as a pseudo-fluid with smoothly varying properties, and the local thermal, compositional, and mechanical equilibrium occurs on the scale of a single grain. Equations (5)–(8) gives the liquid–solid mixture’s properties as weighted averages of the phase-averaged properties. In the analyzed case, the lever-rule model of micro-segregation is used in calculations. The local thermodynamic equilibrium is assumed at the phase interface, so the following relation between solute concentrations holds: k_p_C_L_* = C_S_*, where k_p_ is the equilibrium partition coefficient; C_L_* and C_S_* are the actual solute concentrations at the liquid and solid side of the interface, respectively. According to the assumed lever-rule micro-segregation model, both surface concentrations are equal to volume-averaged ones, namely C_S_ = C_S_* and C_L_ = C_L_*. The parameter k_p_ is lower than 1, so the solubility of the solute is lower in the solid phase than in the liquid. It results in local chemical segregation of elements. The solute is rejected into the liquid phase (micro-segregation) and then transported with inter-dendritic liquid towards the bulk melt (macro-segregation). As a result, solute depletion is observed in the porous zone, while the liquid is enriched in the slurry region.

The hydrodynamic interaction between the molten alloy and the columnar dendrite porous structure is described with the Kozeny–Carman relation

(13) $$K=\frac{\lambda_{2}^{2}}{180}\frac{{\left(1-g_{S}\right)}^{3}}{g_{S}^{2}},$$

under the assumption that the micro-scale parameter, namely the secondary dendrite arm spacing, λ_2_, is constant, and the porous structure is isotropic.

In Equation (2), the solid phase viscosity is defined differently in the slurry and porous zones. In the former, it is determined based on the Ishii and Zuber [39] rheological model, where the slurry dynamic viscosity equals the following: $\mu_{m}={\left(1-g_{S}/g_{S,coh}\right)}^{-2.5g_{S,coh}}\mu_{L}$, and is also a weighted average of solid and liquid viscosities $\mu_{m}=\left(1-g_{S}\right)\mu_{L}+g_{S}\mu_{S}$. In the porous zone, the solid phase velocity equals zero, so the third term on the right-hand side of Equation (2) disappears. In the control volumes close to the interface between the porous and slurry regions, demarcated by the Front (EP-FT model), the switching function, Ṽ, is used to switch between viscosity models smoothly.

On the other hand, in the EP-CP model, the coherency solid fraction isoline is captured, and the coherency switching function, ṼCP, is used to commute between viscosity models smoothly.

The local equilibrium process is assumed, where the hydrodynamic forces between phases are balanced, the locally averaged temperature is uniform at the scale of a single grain, and the steady-state solute transport across the phase boundary is assumed. The equal densities of solid and liquid phases are assumed, so alloy shrinkage-induced solidification is neglected. The cast is assumed to be filled with alloy, molten or solidified, so no free surface is present and the impact of the presence of the free surface on the process is neglected.

Material properties and boundary conditions used in the simulations are gathered in Table 1.

### 2.5. Computer Simulation and Solution Procedures

**Figure 3:**
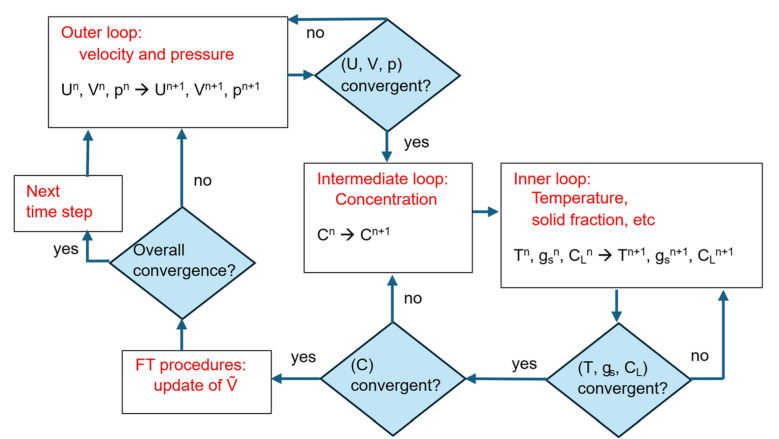
Flowchart of the meso-macroscale numerical model of solidification accounting for the identification of porous and slurry zones.

The EP-CP and the EP-FT computer simulation models for the binary alloy centrifugal casting have been developed and implemented as in-house codes. The mixture mass, momentum, energy, and solute conservation equations; Equations (1)–(4) are supplemented with the closure relations, Equations (5)–(11) have been discretized on a non-orthogonal triangular control-volume mesh, and the implicit Euler scheme has been used for marching in time. The diameter of a single control volume of the nominal mesh is 1 mm, and the corresponding number of control volumes is 12,358. Since the computational mesh is non-orthogonal, the skew diffusion terms are taken into account, where gradients of the field quantities at the control volume’s faces are determined with the cell-based approximation scheme [42]. The collocated mesh concept, where all parameters are stored in a control-volume centroid, has been adopted along with the Rhie and Chow scheme [43] for local velocities at the cell faces, applied to avoid numerically generated spurious wavy modes of the pressure field. The first order upwind scheme [44] has been used for the field variables on the control volume’s faces, and the fractional step computational algorithm [45] has been applied to couple the pressure and velocity fields. In the EP-CP calculations, different grain structures and the associated distinct models of the melt flow resistance within the mushy zone are identified by the DCP model (see Section 2.4). On the other hand, in the EP-FT simulation model at each consecutive time step, after the iterative solving of the conservation equations (Equations (1)–(4)), the front tracking procedure is used. Zones of the columnar and equiaxed grains are recognized based on the most current temperature and concentration fields. Next, the switching function is calculated for each control volume, and the solution procedure is continued at the next step for the updated structure of the mushy zone. At each time step, the three nested iteration loops are executed. The schematic of the simulation procedures organization is presented in Figure 3.

## 3. Credibility Analysis of the Analyzed Computer Simulation Models

### 3.1. Validation of the Model

The proposed EP-FT model was previously validated by comparing calculations with the experiment carried out by Hebditch and Hunt [46], for the case of solidification of the Pb-48wt.% Sn alloy in terrestrial gravity conditions (Seredyński and Banaszek [26]). The model was also verified there, for the side cooling mold of Pb-18wt.% Sn alloy, by comparing the model’s predictions with results of numerical simulations carried out by the EPM-SIMAP and CEMEF groups (Combeau et al. [47]). It was concluded in [26] that although all compared models predicted very similar locations of macro-segregation regions, their results visibly differed in the forecasted numbers and sizes of segregated channels with the enhanced solute concentrations, confirming that numerically predicted channeling is susceptible to the used discretization model.

In this research, the validation analysis of the hybrid enthalpy–porosity models is extended by taking advantage of the experiments conducted in the frame of the AFRODITE project for the selected case of solidification of Sn-10wt.% Pb alloy without electromagnetic stirring, published by Hachani et al. [48,49].

**Figure 4:**
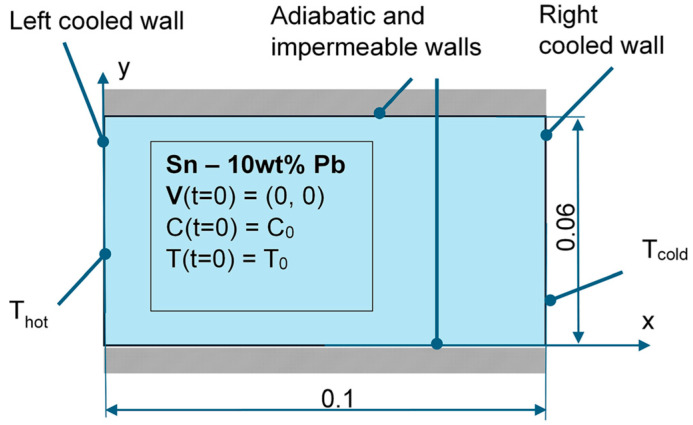
Schematic of the simplified geometry and boundary conditions of the AFRODITE benchmark casting. The casting dimensions are given in meters. The dimension perpendicular to the x-y plane is omitted, the 2D geometry is considered.

The schematic of the analyzed 2D cast is presented in Figure 4 along with the initial and boundary conditions. The impermeable, non-slip, and adiabatic boundary conditions are imposed on both horizontal walls. Both vertical walls are also treated as impermeable and non-slip interfaces, and the temperature boundary condition is defined there. In the AFRODITE experiment, the temperature of both vertical walls was maintained approximately constant for more than 500 s before the cooling stage.

Initially, the constant temperature of 260 °C and composition of 10 wt.% of Pb were imposed in the domain, and the melt velocity was set to zero. Next, the temperature of the right wall dropped to 240 °C while the temperature of the left wall increased to 280 °C. The temperature difference of 40 °C between the cavity vertical walls was maintained until thermal natural convection developed.

After the convective flow developed, the cooling of the right and left walls started, with the same cooling rate, equal to 0.03 K/s, like in the AFRODITE experiment (see, e.g., [48,49]). To enable accounting for the growth of the columnar front from both vertical walls, two moving across the domain front structures were defined, along the left wall and along the right wall.

**Figure 5:**
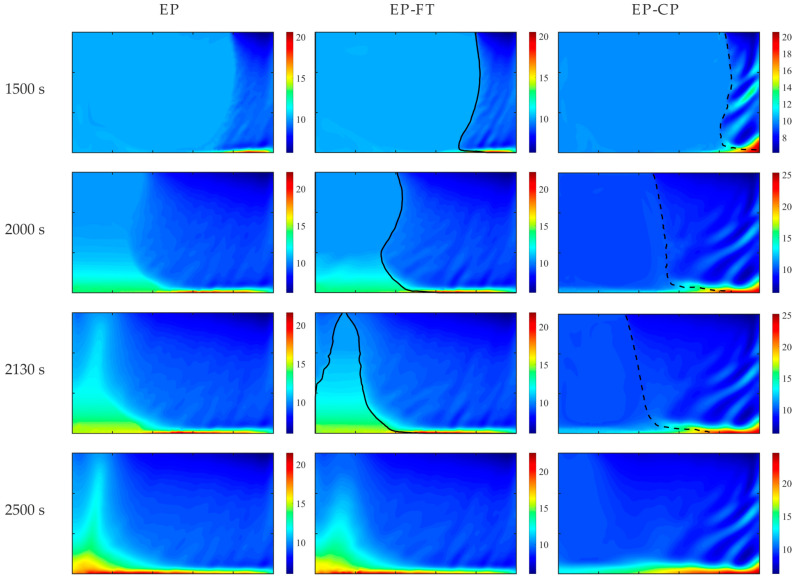
Comparison of solute concentration maps predicted with EP, EP-FT, and EP-CP models, for the selected times of the mold cooling. The casting size is 0.1 m × 0.06 m. The solid and dashed lines shows the location of columnar dendrite tip front and the coherency point solid fraction, respectively. The position of the columnar dendrite tip envelope (EP-FT model) and the isoline of the solid phase fraction, corresponding to the coherence point value of 0.3 (EP-CP model), are marked with a continuous and dashed line, respectively.

The simulations were carried out using the enthalpy–porosity (EP), EP-CP with the coherence point value of 0.3, and the EP-FT models. The distribution of the Pb concentration is compared in Figure 5 for four selected times during the mold cooling and solidification.

**Figure 6:**
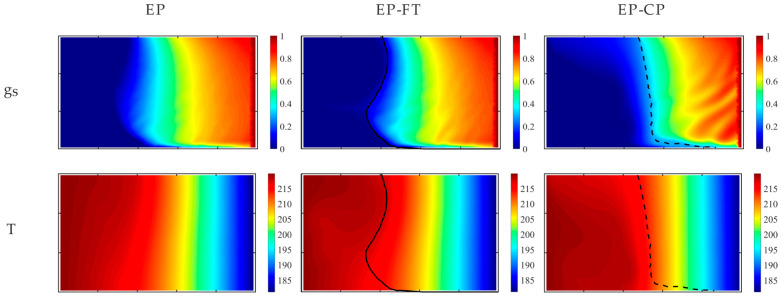
Comparison of solid fraction and temperature predicted with EP, EP-FT, and EP-CP models after 2000 s of the cooling process. The position of the columnar dendrite tip envelope (EP-FT model) and the isoline of the solid phase fraction, corresponding to the coherence point value of 0.3 (EP-CP model), are marked with a continuous and dashed line, respectively.

Figure 6 presents the solid fraction and temperature maps at 2000 s after the onset of the mold’s lateral wall cooling, predicted by the considered models. The separation lines of different dendritic structures in the mushy zone, i.e., the front of the columnar dendrite tips in the EP-FT model and the isoline of the coherence point value of the solid fraction (equal to 0.3), are marked in Figure 5 and Figure 6 with a continuous line and a dashed line, respectively.

The results reveal the formation of the solid phase at the right, cooled wall, and its propagation towards the left side. Segregation channels develop in the right part of the domain. They are enriched in solute liquid agglomerates at the bottom due to the downward orientation of thermal and solutal buoyancy forces for the considered alloy. The solute, solid fraction, and temperature patterns predicted with the EP and EP-FT models are similar but differ from those obtained with the EP-CP model. The segregation channels predicted with the EP-CP model are thicker, and the most enriched in solute region develops at the bottom, close to the colder wall. This difference is more pronounced in the later solidification times (the last row in Figure 5), where a positive segregation region close to the left wall is predicted with EP and EP-FT models only. Also, the formation of two fronts is visible, at time 2130 s (Figure 5), which further merge where there is the most enriched in Pb alloy.

**Figure 7:**
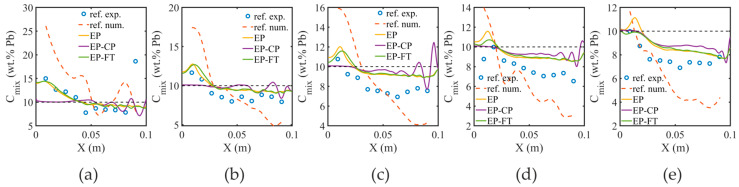
Cross-sections across the domain for the completely solidified cavity, determined at various elevations: (**a**) 1 cm; (**b**) 2 cm; (**c**) 3 cm; (**d**) 4 cm; (**e**) 5 cm; predicted with the EP, EP-CP and EP-FT models and compared to experimental (Hachani et al. [48]—blue dots) and numerical results (Zheng et al. [41]—dashed line). The black dashed line indicates the nominal composition value.

The more detailed comparison of solidification models is presented in Figure 7, where the solute concentration profiles in the completely solidified alloy along the selected horizontal cross-sections are compared with the reference experimental (Hachani et al. [48]) and numerical data (Zheng et al. [41]). The results predicted with the EP and EP-FT models are similar. It can be related to the conditions of solidification, where the rate of motion of the liquidus isotherm and the location of the columnar dendrite tips are reduced by the cooling rate of vertical walls. Also, the vigorous thermal convection is maintained with the high temperature difference between vertical walls, so the distance between the liquidus isoline and the columnar dendrite front is reduced, and as a result, the undercooling zone in front of the columnar dendrite envelope is reduced. The measured solute concentration is clearly higher than that predicted by EP and EP-FT models at each cross-section. In contrast, the reference numerical model (Zheng et al. [41]) predicts more severe macro-segregation than the experimental findings. The EP-CP model results in more pronounced segregation channels, appearing along almost the entire height of the domain as oscillations of the concentration curves.

**Figure 8:**
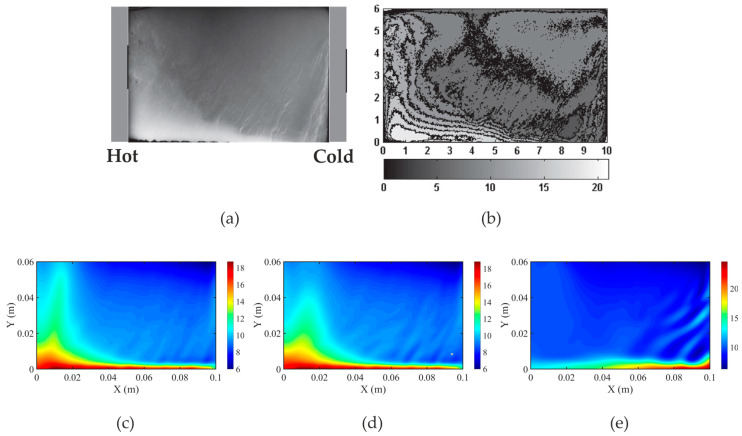
Comparison of solute concentrations in the completely solidified alloy: (**a**) the X-radiography map of Pb concentration [49]; (**b**) its digitally processed picture [49]; (**c**) the EP; (**d**) the EP-FT; (**e**) the EP-CP (g_s,coh_ = 0.3) calculations, respectively. (**a**,**b**) are reprinted from publication [49], with permission from Elsevier.

In Figure 8, the calculated solute concentrations in the completely solidified alloy are juxtaposed with the X-radiography map of Pb concentration (Figure 8a) and its digitally processed picture (Figure 8b) published in [48]. The comparisons confirm a reasonable agreement between the solute macro-segregation and channeling pictures predicted by the EP-FT model and visible discrepancies in the case of the EP-CP calculations. The pictures of segregated channels predicted with the EP and EP-FT models are similar to those determined with the X-radiography imaging method. Also, both models predict the appearance of the macro-segregation region at the bottom of the domain, its expansion towards the left wall, and the formation of the Pb-enriched zone close to the left wall.

### 3.2. Mesh Sensitivity Analysis

Solidification in a rotating reference frame, at elevated gravity accelerations, is extremely difficult to investigate experimentally. So far, few experiments have been carried out in geometries that are not convenient for analyzing the impact of the natural thermo-solutal convection on macro-segregation development. So, due to a lack of an appropriate benchmark experiment accounting for elevated gravity acceleration, the sensitivity analysis has been conducted to confirm the credibility of the proposed EP-FT model.

**Figure 9:**
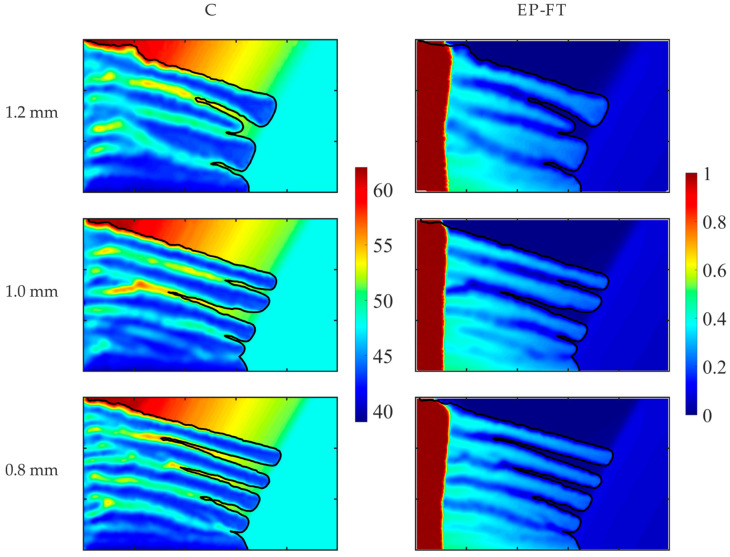
Impact of the mesh density on the Sn concentration (**left** column) and the solid fraction (**right** column) after 250 s of cooling. The casting size is 0.1 m × 0.06 m.

The impact of the mesh density on the solute macro-segregation prediction is presented in Figure 9. The considered case corresponds to the rotating system configuration presented in Figure 1. The effective gravity acceleration, g*, is equal to 5 g, and the rotation angle, β, is 150°. The applied material properties and boundary conditions for the analyzed Pb-48wt% Sn alloy are given in Table 1. Simulations have been conducted with the EP-FT model for three selected mesh densities of unstructured triangular control volumes, of diameters of a single control volume, 1.2 mm, 1.0 mm, and 0.8 mm, and corresponding numbers of control volumes, 8652, 12,358, 19,482, respectively, with the same time step of 0.005 s. In the considered case, where the solutal and thermal buoyancy forces act in opposite directions, the complex structure of the front and the segregation patterns develop (Figure 9). In the upper part of the mold, the enriched liquid is accumulated, which prevents the growth of dendrites close to the top wall, decreasing the local liquidus temperature. In the central part, the development of columnar dendrites is disturbed by the complex thermo-solutal convection, and some macro-scale segregation channels form. Intense convection occurs in front of growing solid structures towards the hot liquid, and the molten alloy is well-mixed there. The qualitative similarities between both predicted parameters, namely solute concentration and solid fraction, are evident. Increasing the mesh density, the front shapes tend towards the one predicted with the densest mesh. The impact of the mesh density is evident because the mesh resolution should be enough to capture the two-way flow inside the channels.

The above mesh sensitivity study confirms that the accuracy of numerical predictions of channel segregation’s details is very prone to the quality of geometrical discretization, so to catch all channeling details, a sufficiently dense mesh should be used. However, having in mind that the main objectives of the paper are to analyze the impact of hyper-gravity’s strength and direction on the developing chemical inhomogeneity in a solidifying alloy, and the comparison of various enthalpy–porosity models’ accuracy for the same geometrical discretization, the control volume mesh of 1.0 mm-diameter has been chosen for further presented calculations. This compromised choice is computationally optimal and allows for capturing the remelting phenomenon in the columnar dendrite zone and the channel segregation pictures with acceptable accuracy.

## 4. Simulation of the Pb-48wt.% Sn Centrifugal Casting—Results and Discussion

To compare the three enthalpy–porosity models’ predictions and to study the influence of the hyper-gravity’s strength and direction on the solute macro-segregation created in the centrifuge, the example of the rotating cavity filled with Pb-48wt.% Sn alloy and cooled from one side (Figure 1) is analyzed. All other cavity walls are kept adiabatic. The effective gravity acceleration, g*, depends on the angular velocity, ω, and the distance from the R-axis. Three values of g* are considered, namely 1 g (for ω = 0 rad/s), 5 g, and 15 g, where g stands for the terrestrial gravity. The second important parameter, which describes the orientation of the cooling direction to the direction of g*, is the β-angle (see Figure 1). The angle changes in the range from 0° for cooling from the bottom to 180° for cooling from the top of the rotating cavity.

### 4.1. Comparison of the EP, EP-CP, and EP-FT Predictions of the Solute Macro-Segregation

The three enthalpy–porosity models have been used to simulate the centrifugal casting of the Pb-48wt.% Sn alloy cooled from one side (Figure 1). All other cavity walls are kept adiabatic.

As discussed above, the main problem in the EP-CP simulation is that the experimentally determined solid fraction at the g_S,coh_ is restricted to only a few alloys (mainly for Al-based ones, see [36,37]). So, it is a common practice to use its arbitrarily chosen values, assuming that it is close to those for Al-based alloys. The EP-CP calculations have been performed for three different values of this critical solid fraction to address the impact of the packing value of solid fraction at the g_S,coh_, on the obtained pictures of Sn concentration.

**Figure 10:**
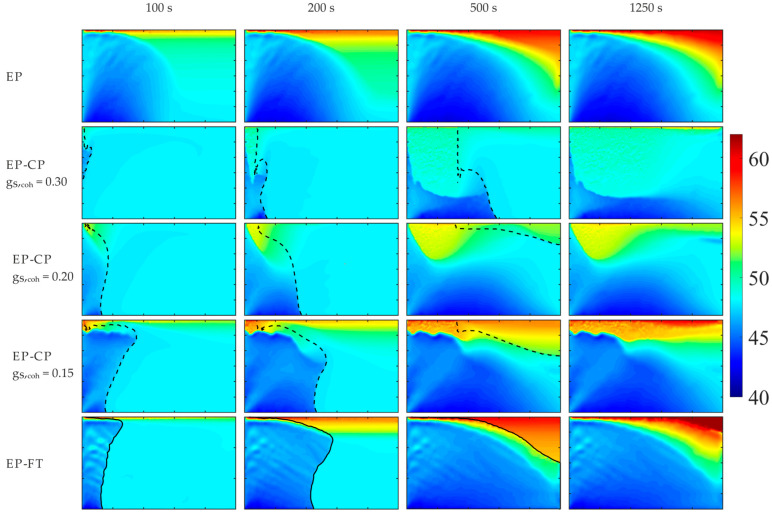
Sn concentration predicted with the EP, EP-CP, and EP-FT models at the selected times of the cooling process for the effective gravity of 1 g and β-angle of 90°. The casting size is 0.1 m × 0.06 m.

The example results are shown in Figure 10, where the evolving Sn concentration is presented for the selected case of β = 90° and the terrestrial gravity, reflecting Hebdich and Hunt’s experiment [46]. The front of columnar dendrite tips in the EP-FT calculations and the isoline of the solid fraction at the coherency point in the EP-CP predictions are presented with the solid and dashed lines, respectively. Both lines split the domain into the columnar porous zone, on the cavity’s cooled wall side, and the solid grains’ slurry, in the hotter region.

The predicted evolving solute segregation pictures and the final distribution of Sn concentration in the fully solidified alloy are evidently dependent on the computer simulation model used. Particularly, the extent of the porous medium is the largest in the EP model, where the whole mushy zone is treated as Darcy’s medium in the form of a stationary, stiff grain matrix.

The solute concentration pictures calculated with the EP-CP model are entirely different (compare the second, third, and fourth rows in Figure 10) for distinct assumed values of the volumetric solid fraction, g_S,coh_, at the coherency point. In the EP-CP simulation, the stationary porous structure forms when the actual local g_S_ exceeds g_S,coh_. Increasing the value of g_S,coh_ results in a higher amount of solid grains present in the undercooled slurry, and the growth of the porous zone is retarded. This observation is supported by comparing different locations of the isoline of constant g_S,coh_ (the dashed line in Figure 10) for three analyzed values of g_S,coh_ at the selected times of the cooling process. This demarcating line, separating the columnar dendrite porous zone from the undercooled melt, moves towards the right wall with lowering values of g_S,coh_.

The predicted zones of macro-segregation and channeling appearance developing during the cooling process and in the completely solidified alloy are visibly dependent on the simulation model. Those obtained with the EP and EP-FT calculations are very different in predicting the porous zone extent and shape, particularly at early times of the process, and some discrepancy in the predicted Sn concentration and channeling pattern is also observed in the completely solidified cast (Figure 10, the right column).

Significant differences in the calculated solute concentration are visible between the EP-CP and the two other analyzed models (Figure 10). These disparities are smaller for a lower value of constant solute fraction adopted at the dendrite coherence point. In the case of g_S,coh_ = 0.15, the final macro-segregation pattern in the completely solidified alloy (the right column in Figure 10) qualitatively resembles the ones obtained from the EP and EP-FT models, predicting the large zone of depletion in Sn concentration at the mold bottom, and the solute-enriched alloy at the top part of the domain, mainly in the right-top corner (the right column in Figure 10).

**Figure 11:**
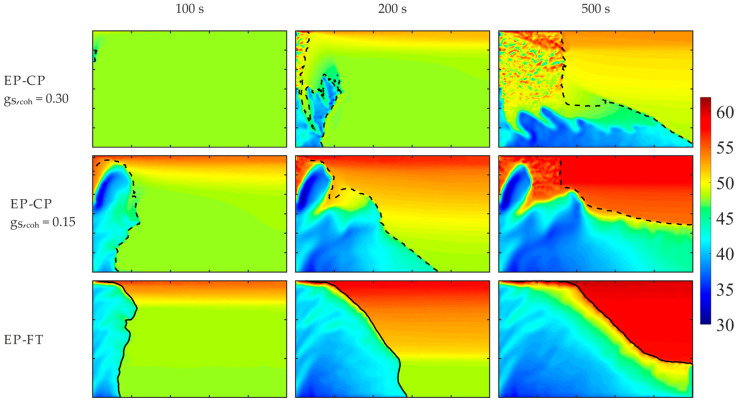
Sn concentration predicted by the EP-CP and the EP-FT, at the selected early times of the cooling process, for the hyper-gravity of 15 g and β-angle of 90°. The casting size is 0.1 m × 0.06 m. The position of the columnar dendrite tip envelope (EP-FT model) and the isoline of the solid phase fraction, corresponding to the coherence point (EP-CP model), are marked with a continuous and dashed line, respectively.

**Figure 12:**
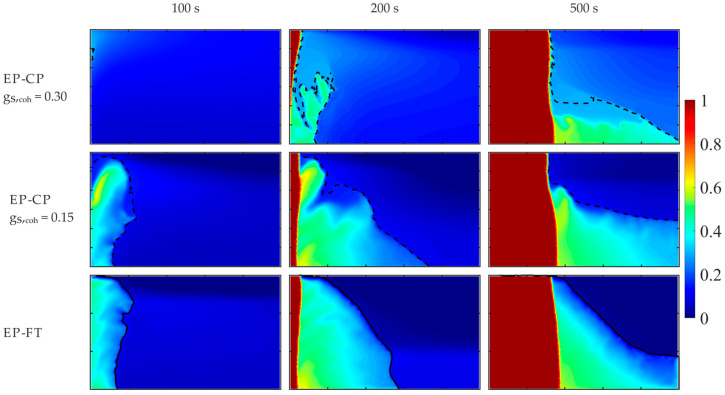
Volumetric solid fraction calculated by the EP-CP and the EP-FT, at the selected early times of the cooling process, for the hyper-gravity of 15 g and β-angle of 90°. The casting size is 0.1 m × 0.06 m. The position of the columnar dendrite tip envelope (EP-FT model) and the isoline of the solid phase fraction, corresponding to the coherence point (EP-CP model), are marked with a continuous and dashed line, respectively.

Further detailed comparisons of the two hybrid enthalpy–porosity models, where the regions of different dendritic structures are distinguished within the mushy zone, i.e., the EP-CP and the EP-FT ones, are given in Figure 11 and Figure 12, where temporal fields of the Sn concentration and the volumetric solid fraction are presented for the enhanced effective gravity of 15 g and β-angle of 90°. For a high g_S,coh_ value, equal to 0.3, formation of the stiff matrix of solid grains is retarded; it appears after 100 s of cooling, while for the other two models, the porous zone is well-developed at this time. Further development of the porous zone results from the complex interplay of convective flow, formation of solid grains in undercooled melt, solidification and remelting, and convective heat transfer.

A zone of merged grains primarily forms in the upper part of the left wall (g_S,coh_ equal to 0.3), and next, it is partially remelted from the top. Later, this structure enlarges from the bottom, where new solid grains transported from the bulk melt are attached to the solid matrix. Above, the local “cavern” appears, filled with the undercooled melt (Figure 12) of the solute at a higher concentration (close to the nominal one) and surrounded by the region of the lower concentration (Figure 11).

Due to convective transport of grains at high solid fraction (g_S,coh_ equal to 0.3), the undercooled liquid is well-mixed in nearly the whole volume, which is evident at time instants 100 s and 200 s. Both other models predict the existence of a well-mixed melt at the bottom and solute stratification at the top. Also, the solid fraction of grains in the melt is visibly higher for the highest g_S,coh_ case than in the other two cases. It causes the later formation of the overheated region at the top in this case (g_S,coh_ = 0.3 at time 500 s, Figure 11 and Figure 12).

Comparison of solute distributions for the same β-angle equal to 90° and various effective gravities, namely 1 g (Figure 10) and 15 g (Figure 11 and Figure 12), enables comparison of the impact of the gravity level on the formation of the porous zone and solute segregation for EP-CP models, g_S,coh_ equal to 0.3 and 0.15, and the EP-FT model. An increase in gravity destabilizes the formation process of the porous zone and promotes the formation of channeling. They are also more evident in the case of the EP-FT model. In the next section, deeper analysis of the impact of the gravity acceleration and cooling direction will be presented.

**Figure 13:**
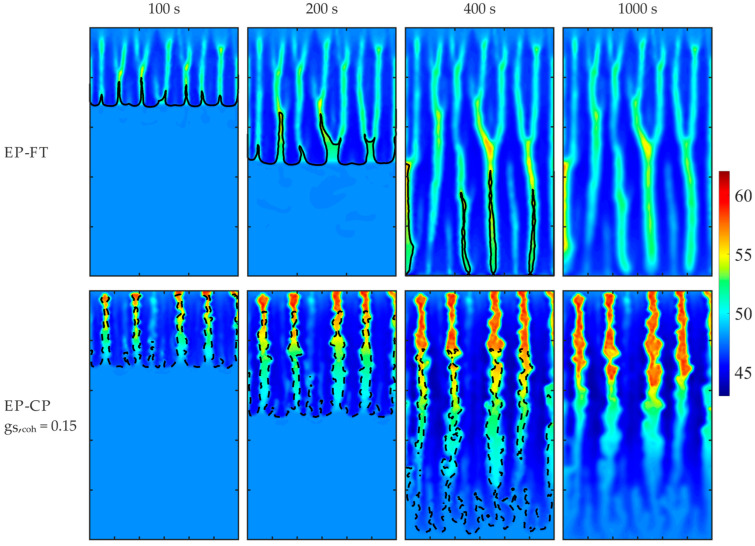
Comparison of Sn concentration predicted by the EP-FT and EP-CP (g_S,coh_ = 0.15) models, for the selected times of mold cooling, in terrestrial gravity and β-angle of 180—the heat is extracted from the mold’s top wall. The casting size is 0.1 m × 0.06 m. The position of the columnar dendrite tip envelope (EP-FT model) and the isoline of the solid phase fraction, corresponding to the coherence point (EP-CP model), are marked with a continuous and dashed line, respectively.

**Figure 14:**
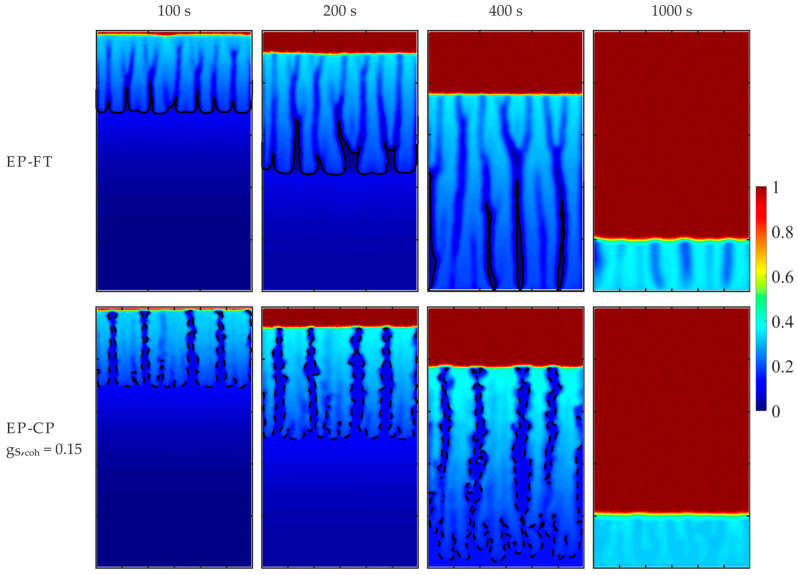
Comparison of the volumetric solid fraction predicted by the EP-FT and EP-CP (g_S,coh_ = 0.15) models, for the selected times of mold cooling, in terrestrial gravity and β-angle of 180–the heat is extracted from the mold’s top wall. The casting size is 0.1 m × 0.06 m. The position of the columnar dendrite tip envelope (EP-FT model) and the isoline of the solid phase fraction, corresponding to the coherence point (EP-CP model), are marked with a continuous and dashed line, respectively.

To further analyze the details of differences between the EP-CP and the EP-FT predictions in the evolution of channel structure, the EP-CP and EP-FT calculations are compared in Figure 13 and Figure 14, where the solute concentration and the volumetric solid fraction are presented for the example case of terrestrial gravity and the rotation β-angle of 180°. The value of the coherency point value was assumed to be 0.15, which gives the closest results to those predicted by the EP-FT model (see Figure 10, Figure 11 and Figure 12). Formation of structures in the form of “fingers” is observed for two cases, but their number is different. The merging and splitting processes are observed in two cases. They cannot be missed with the distinct columnar dendrites, but they are large groups of dendrites, and channels are rather V-channels (V-segregates), which are observed in the central region of castings (e.g., [14]). Also, differences in the evolution of the Sn concentration are evident. The model EP-CP predicts the maximum Sn concentration at the roots of “fingers,” which is close to the maximum available, corresponding to the eutectic composition, so eutectic reaction occurs at the beginning of solidification. For further solidification times, the concentration is reduced along the channels. For the results predicted by the EP-FT model, the Sn concentration increases along channels and does not attain the eutectic level, but is approximately constant along channels at the level 55–56%. The distributions of solid fraction and Sn concentration predicted with the EP-CP model are sharp at the porous/slurry regions boundary, represented with the dashed line. If the EP-FT model is used, the smooth variation in these two fields is observed.

**Figure 15:**
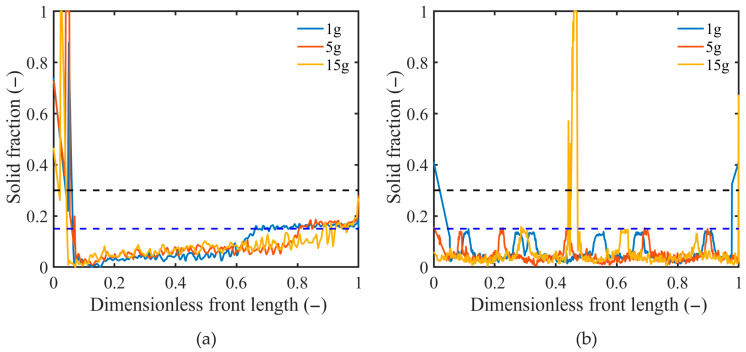
Volumetric solid fraction along the columnar dendrite tips front after 250 s of cooling, for various gravity acceleration levels (1 g, 5 g, 15 g), and β-angles: (**a**) 90° and (**b**) 180°.

**Figure 16:**
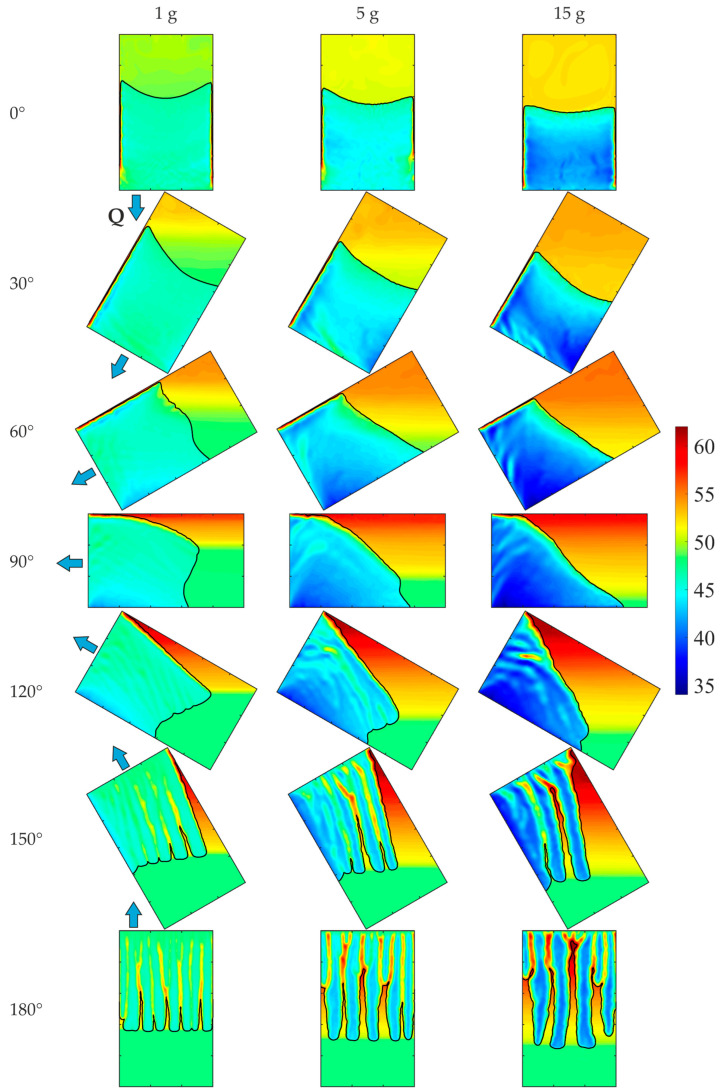
EP-FT predictions of the solute (Sn) concentration after 250 s of cooling for various effective gravity accelerations, g*, and rotation angles, β. The direction of heat transfer through the cooled wall is marked with the blue arrow. The casting size is 0.1 m × 0.06 m.

To further elucidate the impact of the assumed solid fraction at the g_S,coh_ in the EP-CP model on the predicted macro-segregation pictures, changes in the volumetric solid fraction along the columnar dendrite tips front are presented in Figure 15, after 250 s of the mold cooling for various gravity accelerations and two rotation angles, β = 90° and β = 180°. The length of the front (shown in Figure 10, Figure 11 and Figure 12 with the solid black line) is measured starting from the point (x, y) = {0.0, 0.06} in the rotated coordinate system (x, y) (see Figure 1b), and it is normalized to enable comparing various cases. The dashed lines refer to the minimum and maximum coherence point values analyzed in this study. The highest values of the solid fraction (close to 1) are observed in regions where the front motion is stopped due to the presence of the solute-enriched liquid and the decrease in solutal undercooling, so it is captured with the eutectic reaction. It occurs close to the top wall for the angle β = 90° (Figure 10), where the front is nearly motionless and the solute fraction is highest. In the case of β = 180°, the maximum solid fraction appears for the highest gravity acceleration and is close to the central part of the front, corresponding to the deepest channel close to the cooling wall (see Figure 10, the right column). The prevailing values of the solid fraction at the front generally do not exceed the value of 0.15 (blue dashed line), which is attained only close to the well-mixed slurry of solid grains, for β = 90° at the bottom part of the domain (Figure 10 and Figure 15a), and for β = 180°, at the “fingers” tips (Figure 10 and Figure 15a). Impact of the gravity acceleration level on the distribution of the solid fraction along the front is not clear. An increase in the gravity level shifts the region where the solid fraction achieves the value 0.15 to the end of the front, namely to the bottom of the domain (Figure 15a). This behavior is consistent with the reduction in the well-mixed region, where the solid fraction is highest, with an increase in gravity level (see Figure 16). For the case of β = 180° (Figure 15b), the average solid fraction value is nearly the same for all g* values. Locally, the solid fraction increases to the value of 0.15 in locations related to the positions of the tips of “fingers” (see Figure 14). This limiting value is the same for all gravity levels, but the number of these maxima is related to the number of “fingers”.

The above analysis shows that the volumetric solid fraction is not constant but varies along the columnar dendrite front. Therefore, assuming its constant value at the coherence point is questionable for distinguishing regions of different dendritic structures.

### 4.2. The Role of Centrifugal Forces in the Developing Solute Macro-Segregation

Figure 16 presents the EP-FT predictions of the macro-segregation in the solidifying Pb-48wt.% Sn alloy, in terms of the Sn concentration pictures after 250 s of cavity cooling for various effective gravity accelerations and rotation angles. Thorough analysis of these results confirms a significant impact of both the strength and direction of centrifugal forces on the concentration distribution of the alloy’s components and the position and shape of the front separating different dendritic structures. From Figure 16 (rotation angles 0° and 30°), one can conclude that the region of almost uniform solute distribution in the slurry zone rises with the increased effective gravity. The shape and size of the porous region strongly depend on the strength and rotation angle. In this region, more severe channeling is observed in hyper-gravity.

The solute-enriched liquid is accumulated at the top part of the domain, opposite the direction of the effective gravity acceleration vector. Its volume rises with the increasing g*, retarding the further growth of the columnar dendrites and stopping the motion of the dendrite tips front. Stratification of the solute fraction in the molten alloy is seen in the gravity vector direction for almost all considered angles, except for the 0° angle, where the liquid is well-mixed in its whole volume. The extent of the solute stratification region is roughly bounded by the most protruding part of the front, and its size decreases with increasing g*. Due to the enhanced buoyancy forces, the dendrite growth rate rises, and the zone of stationary columnar grains decreases. In contrast, the inclination angle of the front at the top of the cavity increases with the higher values of g*. A-segregation channels formation is observed close to the cooled wall for β angles from 30° to 120°. Their number and size increase with the enhanced gravity acceleration. The other kind of channels oriented perpendicularly are visible for angles from 60° to 180°. Their number decreases with the β angle, but the size (width) of the channels increases with both the β angle and the enhanced gravity g*.

## 5. Final Conclusions

The presented research is the authors’ contribution to the development of advanced meso-macroscopic computer simulations for more accurate predictions of the solute macro-segregation and channeling phenomena under hyper-gravity, where the enhanced mass and heat transfer processes are more complex and challenging than under terrestrial gravity. Macro-segregation is a dynamic process that begins early in the solidification process and progresses over time. The primary goal of computationally efficient meso-macroscopic simulations, based on a single-domain enthalpy–porosity model coupled with the mushy zone models, is to provide valuable data on temporal changes in temperature, solid fraction, and solute concentration within a whole cast domain.

The paper addresses the issue of the role of the assumed model of mushy zone in predicting reliable pictures of solute macro-segregation in hyper-gravity. A simplified 2D model of the representative rotating plane has been developed, based on a thorough analysis of all forces acting on a cast in the centrifuge and their projections onto the plane. It has been used in a detailed comparative study of three different models of flow resistance in the mushy zone, i.e., EP, EP-CP, and EP-FT, when applied to simulate the solidification of Sn-10wt%Pb and Pb-48wt.% Sn alloys under terrestrial and hyper-gravity conditions. The study’s outcomes, in terms of predicted temporal fields of solute concentration and solid fraction, lead to the following conclusions.

- The obtained pictures of macro-segregation and local channeling strongly depend on the mesoscopic model used. The differences in the models’ results increase with the rise in the hyper-gravity strength.
- The columnar porous zone size predicted by the EP simulation is overestimated, particularly at early times during mold cooling, as a consequence of treating the whole mushy zone as a Darcy porous medium.
- The EP-CP calculations are highly sensitive to the assumed coherence value of the solid fraction and provide completely different pictures of the solute inhomogeneity, with some complex structures of merged solid grains (see Figure 11 and Figure 12), which are not present in calculations with a lower coherence point. A critical problem in this popular EP-CP modeling is the lack of experimentally determined solid fraction values at the coherency point for most alloys, except for Al-based ones. Moreover, since the volumetric solid fraction varies in time, along the columnar dendrite front (see Figure 15), and with the hyper-gravity strength and direction, it is disputable to use its constant value at the coherence point for distinguishing regions of different dendritic structures.
- Therefore, the proposed EP-FT model is the most reasonable choice for a reliable simulation of complex macro-segregation pictures, particularly in centrifugal alloy casting, where the melt flow, solute mass, and heat transport processes are significantly enhanced, influencing the evolving grain structure.

The EP-FT model has been positively validated by comparing its results with the AFRODITE experimental benchmark of Sn-10wt%Pb alloy solidification in terrestrial gravity. And, its accuracy has been verified through mesh sensitivity analysis in the case of Pb-48wt%Sn alloy solidification in the centrifuge with 5 g effective gravity acceleration. Then, the model has been used to frame correlations between hyper-gravity forces and the resulting chemical inhomogeneity of the centrifugal casting product. The results presented in the paper confirm the significant impact of both the strength of hyper-gravity and the angle between the cooling direction and the enhanced gravity direction on the solute concentration distribution, as well as the position and shape of the front separating different dendritic structures and the number and size of locally forming highly solute-rich channels.

Our future activity will be oriented towards developing a 3D parallelized simulation of centrifugal casting, accounting for Coriolis forces and built on unstructured adaptive meshes, for more precise modeling of the front separating different dendritic structures and simulations in more complex cast domains. Additionally, the impact of the relative density of solid grains and molten alloy, as well as the role of nominal alloy composition and the related direction of thermal and solute buoyancy forces, will be studied.

## Figures and Tables

**Table 1:** Material properties and boundary conditions used in calculations.

	Symbol	Units	Pb-48 wt.% Sn [40]	Sn-10 wt.% Pb [41]
Initial mass fraction	C_0_	wt%^−1^	48.0	10.0
Density	ρ	kg·m^−3^	9000.0	7000.0
Specific heat	c_L_, c_S_	J·kg^−1^K^−1^	200.0	260.0
Thermal conductivity	k_L_, k_S_	W·m^−1^K^−1^	50.0	55.0
Viscosity	µ	kg·m^−1^·s^−1^	10^−3^	10^−3^
Liquid thermal expansion coefficient	β_T_	K^−1^	10^−4^	6.0 × 10^−5^
Liquid solutal expansion coefficient	β_C_	wt.%^−1^	4.5 × 10^−3^	−5 × 10^−3^
Latent heat	L	J·kg^−1^	53,550	61,000
Diffusion coefficient (liquid)	D_L_	m^2^·s^−1^	1 × 10^−9^	4.5 × 10^−9^
Diffusion coefficient (solid)	D_S_	m^2^·s^−1^	1 × 10^−13^	1 × 10^−12^
Melting temperature of a solvent	T_M_	K	600.65	505.15
Liquidus slope	m_l_	K·wt.%^−1^	−2.334	−1.286
Equilibrium partition coefficient	K	−	0.307	0.0656
Eutectic temperature	T_E_	K	456.15	456.15
Secondary dendrite arm spacing	λ_2_	µm	40.0	65.0
Boundary conditions:				
Initial temperature	T_0_	°C	216.0	−
Temperature of an ambient fluid	T_amb_	°C	25.0	−
Heat transfer coefficient	h	W·m^−2^·K^−1^	400.0	−

## Data Availability

The original contributions presented in this study are included in the article. Further inquiries can be directed to the corresponding author.

## Abbreviations

Notation	Meaning
c	Specific heat, J·kg^−1^·K^−1^
C	Concentration of chemical species, i.e., mass fraction
D	Solute mass diffusivity, m^2^·s^−1^
f	Phase volumetric fraction, -
**F**	Force, N
g	Phase mass fraction, -
**g**	Gravity acceleration, m·s^−2^
h	Heat transfer coefficient, W·m^−2^·K^−1^
k	Thermal conductivity, W·m^−1^·K^−1^
K	Permeability of the porous medium, m^2^
k_p_	Equilibrium partition coefficient, -
L	Latent heat of fusion, J·kg^−1^
m_l_	Liquidus slope, K·wt.%^−1^
**R**	Distance of the gondola from the axis, m
T	Temperature, K
T_E_	Eutectic temperature, K
T_M_	Melting temperature of a solvent, K
**V**	Velocity vector, m·s^−1^
**Ṽ**	Switching function value, -
α	Rotation angle of the gondola, rad
β	Rotation angle of a sample, rad
β_C_	Liquid solutal expansion coefficient, wt.%^−1^
β_T_	Liquid thermal expansion coefficient, 1/K
λ_2_	Secondary dendrite arm spacing, m
*µ*	Viscosity, kg·m^−1^·s^−1^
*ρ*	Density, kg·m^−3^
ω	Angular velocity, rad·s^−1^
Subscript	Meaning
cf	Centrifugal
coh	Coherency
C	Coriolis
g	Gravitational
L	Liquid fraction
m	Mixture
r	Resulting
ref	Reference value
S	Solid fraction
